# Advancements in the Extraction, Characterization, and Bioactive Potential of Laminaran: A Review

**DOI:** 10.3390/foods14101683

**Published:** 2025-05-09

**Authors:** Kit-Leong Cheong, Amanullah Sabir, Min Wang, Saiyi Zhong, Karsoon Tan

**Affiliations:** 1 Guangdong Provincial Key Laboratory of Aquatic Product Processing and Safety, College of Food Science and Technology, Guangdong Ocean University, Zhanjiang 524088, China; klcheong@gdou.edu.cn (K.-L.C.); amaanullahsabir@gmail.com (A.S.); 2 College of Coastal Agriculture Sciences, Guangdong Ocean University, Zhanjiang 524088, China; 3 Guangxi Key Laboratory of Beibu Gulf Biodiversity Conservation, Beibu Gulf University, Qinzhou 535011, China

**Keywords:** laminaran, extraction, purification, characterization, mucosal barrier

## Abstract

Laminaran, a bioactive β-glucan derived from brown algae, has garnered significant attention due to its diverse pharmacological properties, including antioxidant, immunomodulatory, and mucosal protective effects. Despite promising research highlighting its potential applications in functional foods, nutraceuticals, and pharmaceuticals, the commercial utilization of laminaran remains limited, primarily due to challenges in extraction efficiency, structural complexity, and a lack of standardized methodologies. This review critically examines recent advancements in the extraction, purification, structural characterization, and biological evaluation of laminaran. Both conventional and emerging extraction methods—including ultrasound-assisted extraction, microwave-assisted extraction, and enzymatic techniques—are evaluated for their efficiency, scalability, and sustainability. Analytical tools, such as high-performance liquid chromatography, nuclear magnetic resonance, and mass spectrometry, are discussed for their roles in elucidating key structural features, such as molecular weight, degree of polymerization, and glycosidic linkage patterns, which are closely tied to laminaran’s biological activity. Innovative extraction technologies have improved yield and purity, while structural insights have deepened the understanding of structure–function relationships. Interdisciplinary collaboration will be critical to advance laminaran from a marine-derived polysaccharide to a commercially viable bioactive compound for health, nutrition, and biomaterial applications.

## 1. Introduction

Marine polysaccharides are intricate carbohydrates obtained from marine sources such as seaweeds, microalgae, and marine invertebrates. These compounds have drawn considerable interest due to their active biological properties and promising applications in various areas, including pharmaceuticals, nutraceuticals, cosmetics, and biotechnology [1,2,3]. Notable marine polysaccharides—such as alginate, carrageenan, chitin, chitosan, fucoidan, laminaran, and ulvan—possess distinct structural characteristics that contribute to their bioactivity [4,5,6]. They exhibit a broad spectrum of biological effects, including immunomodulatory, antioxidant, antimicrobial, anticoagulant, anticancer, and anti-inflammatory activities [7,8]. The bioactivity of these polysaccharides is influenced by various factors, such as molecular weight, functional groups, branching patterns, and monosaccharide composition [9,10]. Due to their natural abundance and growing recognition in biomedical research, marine polysaccharides are being actively investigated for their potential in drug formulation, wound healing, tissue engineering, functional foods, and cosmetic applications [11,12,13]. Their ability to regulate biological processes with minimal toxicity further highlights their significance in health and medical sciences [14].

**Figure 1:**
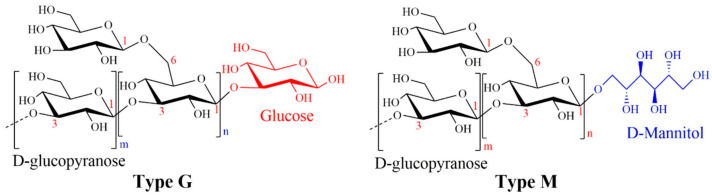
The molecular structure of laminaran, highlighting type G and type M. Laminaran primarily consists of β-1,3-D-glucopyranose units, with β-1,6-linked branches forming the inter-chain backbone. The figure was adapted with permission from reference [23], copyright 2024, Elsevier.

Laminaran, a bioactive polysaccharide primarily found in brown algae (Phaeophyceae), has gained particular attention [15]. It is mainly extracted from seaweeds such as *Eisenia bicyclis*, *Laminaria digitata*, *Laminaria japonica*, *Saccharina latissima*, and *Sargassum henslowianum* [16,17,18]. Structurally, laminaran is a low-molecular-weight β-glucan primarily composed of β-(1→3)-linked D-glucose units with occasional β-(1→6) branches [19,20]. Its molecular weight generally falls between 2 and 40 kDa [21,22]. Laminaran exists in two main forms: the water-soluble G-type and the slightly insoluble M-type, distinguished by the presence or absence of a terminal mannitol group (Figure 1) [23]. Extensive studies have explored its diverse biological functions, including immunomodulatory, antioxidant, anticancer, antimicrobial, and anti-inflammatory properties [22,24,25,26,27]. While a variety of extraction and purification techniques have been investigated, few studies have systematically compared their efficiency, scalability, or suitability for industrial applications. Similarly, analytical approaches including chromatography and spectroscopy have been employed in characterization, yet a standardized analytical framework for laminaran characterization remains lacking. These limitations impede consistency, reproducibility, and the successful translation of laminaran-based innovations to industrial use. Diverse research topics discussing laminaran, ranging from novel extraction technologies to functional food applications, indicate that laminaran is increasingly recognized as a promising marine-derived functional ingredient.

This review presents a comprehensive analysis of laminaran, emphasizing its extraction, purification, structural characterization, and biological activities. By critically evaluating and consolidating current methodologies, we aim to identify optimal strategies that enhance yield and purity and propose viable pathways for industrial-scale implementation. Particular attention is given to structural parameters—such as the molecular weight, degree of polymerization (DP), and branching configurations—and their influence on bioactivity. The novelty of this review lies in its integrative approach, bridging technical processes and functional applications to promote the development of laminaran as a commercially viable marine-derived bioactive compound. This work advances both the academic understanding and practical applications of marine polysaccharides in health, nutrition, and biomaterials.

## 2. Extraction of Laminaran

Effective extraction techniques are crucial for obtaining laminaran with high purity, an optimal yield, and strong bioactivity. Laminaran is widely utilized in pharmaceuticals, cosmetics, and functional foods due to its antioxidant, immunomodulatory, and anti-inflammatory benefits [21,22]. However, extracting laminaran is complex as the cell walls of brown seaweed contain other polysaccharides, such as fucoidan and alginate, which can hinder its isolation [28]. Poor extraction methods may result in degradation, reduced yield, or contamination, ultimately diminishing the biological effectiveness of the final product [29].

Choosing an optimal extraction method is key to ensuring both the environmental sustainability and cost-effectiveness of laminaran production. Laminaran is generally insoluble in organic solvents such as methanol, ethanol, and ethyl acetate, as demonstrated by a mass spectrometer analysis [30]. One of the most commonly employed techniques is hot water extraction (HWE), valued for its simplicity and cost efficiency. This technique utilizes high-temperature water treatment over a controlled period to disrupt seaweed cell walls and extract water-soluble polysaccharides. Garcia-Vaquero et al. optimized HWE at 120 °C for 80.9 min using 12.02 mL/g of *Laminaria hyperborea*, achieving a yield of 2344.1 mg per 100 g of dry seaweed [31]. Likewise, extracting *Durvillaea potatorum* under optimized acidic conditions (0.05 mol/L HCl at 60 °C for 3 h) resulted in a higher total polysaccharide yield (43.57%, *w/w*), including laminaran and fucoidan [32]. HWE continues to be widely used due to its scalability and minimal reliance on harsh chemicals, making it a more environmentally friendly approach. However, its drawbacks include prolonged extraction times and high energy demands at elevated temperatures, which can reduce overall efficiency [33].

Innovative extraction techniques, including ultrasound-assisted extraction (UAE), microwave-assisted extraction (MAE), and enzymatic-assisted extraction (EAE), provide efficient alternatives to conventional methods for obtaining laminaran with improved yield and purity [34]. These approaches enhance extraction efficiency, shorten processing time, and boost overall yield compared to traditional techniques [35]. Examples of laminaran extraction are presented in Table 1.

UAE leverages high-frequency sound waves to create microbubbles that disrupt cell walls, promoting laminaran release while improving energy efficiency. When conducted at 60% ultrasonic power amplitude with 0.1 mol/L hydrochloric acid for 15 min, UAE achieved laminaran yields of 5.82% and 6.24% (dry weight basis) from *Ascophyllum nodosum* and *Laminaria hyperborea*, respectively [36]. Similarly, response surface methodology-optimized UAE, performed at 500 W nominal power, 20 kHz frequency, 60 °C, and 100% ultrasonic amplitude for 10 min, resulted in a total laminaran content of 1014.4 mg per 100 g of dried *Laminaria digitata* [29]. UAE offers advantages such as a reduced extraction time, enhanced yield, and minimal solvent usage. However, its limitations include limited penetration depth in dense materials and the need for specialized equipment [37].

MAE utilizes rapid volumetric heating through the dielectric polarization of water molecules, which accelerates cell wall rupture and enhances the solubilization of laminaran. For example, MAE conducted at 80 W for 15 min with a solid-to-liquid ratio of 1:10 (*w/v*) successfully extracted laminaran from *Sargassum ilicifolium* [38]. The technique is known for its quick extraction times and high yields. However, it has limitations, including uneven heating and the requirement for specialized equipment [39].

EAE employs specific enzymes to selectively break down the seaweed cell wall, allowing for the preservation of laminaran’s bioactivity while minimizing contamination from other polysaccharides. The optimization of EAE using the cellulase Celluclast^®^ under conditions of 60 °C, pH 4.0, and an enzyme-to-substrate ratio of 4.0% (*v/dw*) resulted in a yield of approximately 57% from *Ecklonia maxima* [40]. These advanced extraction methods not only enhance efficiency but also promote environmental sustainability by reducing energy consumption and minimizing solvent waste. However, some limitations include the high cost of enzymes, potential scalability issues, and the risk of bioactivity loss due to excessive enzyme usage or degradation [41].

The extraction of laminaran has seen continuous advancements, with growing emphasis on eco-friendly and sustainable approaches that minimize environmental impact while improving extraction efficiency. HWE remains widely employed at the industrial scale due to its simplicity, cost-effectiveness, and ease of operation. In contrast, advanced extraction techniques—UAE, MAE, and EAE—are predominantly utilized at the laboratory scale. These methods, although offering enhanced yield and selectivity, often require specialized equipment and process optimization, which currently limits their large-scale implementation. Future research should prioritize the optimization of advanced extraction techniques, such as UAE, MAE, EAE, which can improve yield while reducing waste and reliance on chemical solvents. However, scaling these techniques for industrial applications remains a key challenge. Addressing factors such as cost-effectiveness, scalability, and process standardization is essential to ensure commercial viability. Incorporating automation, continuous-flow extraction systems, and hybrid methodologies may help overcome these limitations, leading to greater efficiency, consistency, and productivity. For instance, ultrasonic energy can be continuously applied to a flowing stream of seaweed slurry. This process is optimized through the integration of sensors and control systems that monitor key parameters such as temperature, flow rate, ultrasonic intensity, and extraction duration in real time. Hybrid methodologies that combine multiple techniques—such as the integration of UAE with MAE—offer synergistic advantages by improving cell wall disruption, enhancing solvent penetration, and significantly reducing overall extraction time.

## 3. Purification of Laminaran

Precipitation techniques are commonly employed for laminaran purification due to their ease of use, affordability, and scalability. Among these, ethanol precipitation is the most widely utilized method, typically involving 70–80% ethanol to isolate laminaran while excluding smaller contaminants such as proteins, salts, and low-molecular-weight carbohydrates. However, this approach alone is often inadequate for achieving complete purification. A prior study demonstrated the extraction of crude laminaran from *Sargassum henslowianum* using ethanol precipitation [17]. Additionally, ethanol precipitation can aid in the removal of unhydrolyzed polysaccharides, resulting in a supernatant enriched with laminaran oligosaccharides [42]. Although precipitation offers a simple means of purification, supplementary processes such as dialysis or chromatography are frequently necessary to eliminate residual solvents and improve purity. Despite these constraints, precipitation remains a fundamental initial step, especially in large-scale industrial applications. For industrial-scale operations, the extraction process can be adapted using continuous or batch reactors equipped with precise temperature control, efficient agitation systems, and automated ethanol dosing. At this scale, efficient ethanol recovery and recycling are essential for ensuring cost-effectiveness and reducing environmental impact. Additionally, safety becomes a critical concern, requiring strict compliance with industrial safety regulations and protocols to mitigate risks associated with solvent handling, flammable materials, and high-temperature conditions.

Chromatographic methods, such as ion-exchange chromatography and size-exclusion chromatography, are essential for laminaran purification, allowing precise separation from co-extracted polysaccharides, proteins, and other impurities to achieve high purity [43]. Ion-exchange chromatography is particularly effective in distinguishing laminaran from charged polysaccharides, proteins, and ionic contaminants in crude extracts [44]. Given that laminaran is generally neutral or slightly anionic, anion-exchange chromatography is commonly utilized. Negatively charged impurities, including fucoidans, alginates, and proteins, adhere to the positively charged resin, whereas laminaran remains unbound or elutes with a mild salt gradient [14]. A study demonstrated that laminaran extracted from *Dictyota dichotoma* was purified using DEAE-cellulose, where the neutral laminaran fraction was eluted with water. Meanwhile, mannuronic acid and fucoidan with a low degree of sulfation were eluted with 1 mol/L of NaCl, while highly sulfated fucoidan fractions required 2 mol/L of NaCl [45]. Similarly, polysaccharides from *Sargassum duplicatum*, containing both neutral and ionic polysaccharides, were purified using a DEAE-cellulose column pre-equilibrated with 0.1 mol/L of HCl, with laminaran being eluted using water [46]. While ion-exchange chromatography provides high specificity and results in highly purified laminaran, it is frequently combined with other techniques, such as size-exclusion chromatography or membrane separation, for enhanced purification.

Size-exclusion chromatography, which separates molecules based on size differences, is another effective approach for purifying laminaran. This technique employs a porous gel matrix, allowing smaller molecules to penetrate deeper and elute later, while larger molecules pass through more quickly [47]. Size-exclusion chromatography is particularly advantageous for isolating laminaran from smaller carbohydrates and low-molecular-weight contaminants present in crude extracts. The selection of gel filtration media is crucial for optimizing separation efficiency based on laminaran’s molecular weight distribution [48]. Although size-exclusion chromatography allows for high-resolution separation without the need for harsh solvents or chemical modifications [49], its drawbacks include a lower sample loading capacity and extended processing times. For instance, one study reported the purification of a 500 mg polysaccharides fraction using a Sepharose 6B column eluted with 0.1 mol/L NaCl, followed by further purification with a Sephacryl S-300 HR column, ultimately yielding 17.5 mg of highly purified laminaran (3.5% of the initial purified fraction) [17].

Membrane-based methods, including ultrafiltration and dialysis, provide an effective and scalable approach for purifying laminaran by eliminating low-molecular-weight contaminants such as salts and small metabolites [50]. Ultrafiltration employs semipermeable membranes with specific molecular weight cutoffs to selectively retain laminaran while allowing smaller impurities to pass through [51]. This technique is particularly advantageous for concentrating laminaran solutions and separating different molecular weight fractions of the polysaccharide. For instance, Ummat et al. processed extracts from *Fucus vesiculosus* and *Ascophyllum nodosum* using an ultrafiltration membrane, where high-molecular-weight fucoidan was retained in the retentate, while low-molecular-weight laminaran was recovered from the permeate [52]. In contrast, dialysis involves placing the laminaran solution in membrane tubing with a defined molecular weight cutoff and submerging it in a large volume of deionized water or buffer. This enables the gradual removal of unwanted small molecules through passive diffusion. Dialysis is frequently employed as a post-ion-exchange chromatography step to eliminate residual salts, buffer components, and small ionic impurities that may persist in the laminaran solution after elution [53,54].

**Figure 2:**
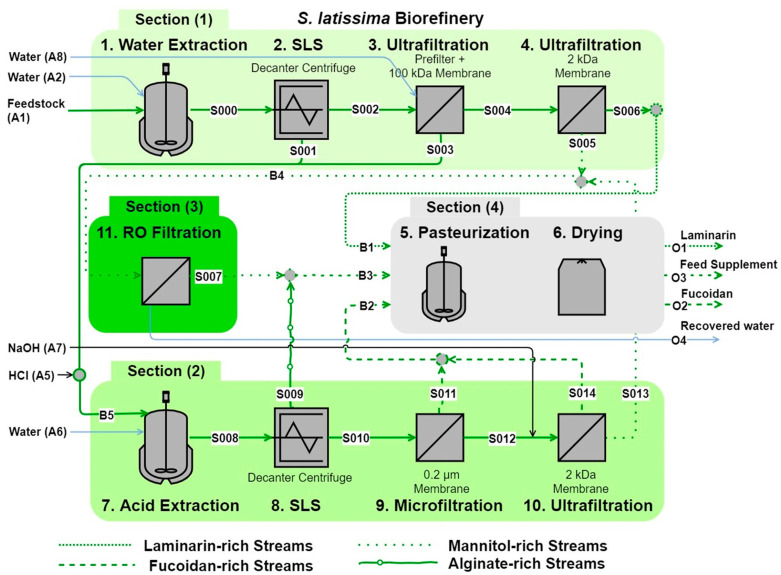
Industrial-scale membrane-based purification of laminaran. This flow diagram illustrates the key processing steps, inputs, outputs, and process streams involved in the purification of *Saccharina latissima*. The extraction process passes through ultrafiltration membranes with both high and low molecular weight cutoffs to separate larger-molecular-weight components, such as fucoidan and alginate, while concentrating laminaran. SLS: solid–liquid separation; RO: reverse osmosis. Adapted with permission from reference [56], copyright 2024, Elsevier.

Membrane-based techniques offer a simple, cost-effective, and chemical-free approach, making them a viable option for the large-scale purification of laminaran [55]. For example, a previous study employed hydrothermal extraction followed by ultrafiltration using a high-molecular-weight cutoff membrane. This step retained larger polysaccharides, such as fucoidan and alginate, while allowing lower-molecular-weight laminaran to pass through. In a subsequent stage, laminaran was further concentrated using an ultrafiltration membrane with a lower molecular weight cutoff (as illustrated in Figure 2) [56]. On an industrial scale, this method was implemented to standardize feedstock processing rates across various systems, handling 5000 kg of dry matter per batch—equivalent to 900 metric tons annually. This approach was successfully applied to *Fucus vesiculosus*, *Laminaria digitata*, and *Saccharina latissima*, yielding 1050, 1043, and 974 metric tons of total processed weight, respectively, as fine dry powders [56].

**Table 1:** Extraction methods, characterization techniques, and biological activities of laminaran derived from different brown seaweed species.

Species	Extraction Method	Characterization Technique	Biological Activity	Ref.
*Ascophyllum nodosum*	Ultrasound assisted	MALDI-TOF MS	Antioxidant	[36]
*Laminaria digitata*	Ultrasound assisted	Colorimetric	Antioxidant	[29]
*Laminaria hyperborea*	Thermal	HPLC-MS	-	[30]
*Laminaria japonica*	-	HPLC	Anticancer	[16]
*Padina pavonica*	Thermal	MALDI-TOF MS	Anticancer	[54]
*Saccharina latissima*	Thermal	NMR	-	[57]
*Sargassum ilicifolium*	Microwave assisted	HPLC	Antioxidant, antimicrobial	[38]
*Undaria pinnatifida*	Thermal	HPLC, NMR	Mucosal barrier	[53]

MALDI-TOF MS: matrix-assisted laser desorption/ionization time-of-flight mass spectrometry; HPLC: high-performance liquid chromatograph; NMR: nuclear magnetic resonance.

## 4. Qualitative and Quantitative Analyses of Laminaran

### 4.1. Colorimetric Method

A colorimetric method is a widely used analytical technique for the quantitative determination of laminaran, relying on color changes that occur when the polysaccharide reacts with specific chemical reagents.

One of the most commonly used colorimetric assays for polysaccharide quantification is the phenol–sulfuric acid method. This technique involves the reaction of laminaran with concentrated sulfuric acid and phenol, resulting in the formation of a colored complex that exhibits maximum absorbance at approximately 490 nm [58]. For instance, Jayapala et al. quantified the content of laminaran oligosaccharides using this method [42]. Alternatively, Caballero et al. utilized the 3-methyl-2-benzothiazolinone hydrazone colorimetric method to determine the laminaran content in *Phaeodactylum tricornutum*, measuring absorbance at 620 nm [59]. Colorimetric methods are simple, low cost, and available for the high-throughput screening of the carbohydrate content in various biological and environmental samples. However, they lack specificity as they respond to all carbohydrates present in a sample, making them less reliable when distinguishing laminaran from other polysaccharides.

### 4.2. Thin-Layer Chromatography

Thin-layer chromatography (TLC) is a widely used technique for analyzing low-molecular-weight laminaran and its derived oligosaccharides, allowing for the separation and identification of carbohydrate components based on their mobility on a stationary phase. This method has been utilized to examine low-molecular-weight laminaran, including oligosaccharides with a DP ranging from 2 to 9 [42]. TLC offers a fast and cost-efficient approach for monitoring laminaran degradation, assessing enzymatic hydrolysis, and detecting various oligosaccharides in complex mixtures. For example, a previous study employed TLC for the preliminary identification of the hydrolytic activity of β-glucanases (Cgglu17A and Cgglu16B). These enzymes, derived from *Chaetomium globosum* W7, were confirmed to degrade the cell walls of *Fusarium sporotrichioides*, yielding laminaribiose, laminaritriose, laminaritetraose, and laminaran [60].

### 4.3. High-Performance Liquid Chromatography

High-performance liquid chromatography (HPLC) is a widely used technique for analyzing laminaran, offering high resolution, sensitivity, and reproducibility in the separation and quantification of its molecular components. In HPLC analysis, laminaran and its hydrolysis products, such as oligosaccharides, are separated based on their polarity or molecular size using different chromatographic columns, including reversed-phase chromatography, hydrophilic interaction liquid chromatography (HILIC), and size-exclusion chromatography. Reversed-phase chromatography is commonly applied to determine the monosaccharide composition of laminaran. This process involves pre-column derivatization with phenyl-3-methyl-5-pyrazolone, followed by separation on a reversed-phase column. Analysis has shown that purified laminaran predominantly consists of glucose [61]. Graiff et al. utilized HPLC to measure laminaran contents in brown seaweed and discovered that *L. hyperborea* and *L. digitata* had notably high concentrations, approximately 8.58 and 7.51 mg/g of dry weight, respectively. Conversely, *Saccharina latissima*, *F. serratus*, and *F. vesiculosus* exhibited lower laminaran contents, ranging from 0.12 to 0.93 mg/g of dry weight [30]. HILIC, on the other hand, is used for the separation of laminaran oligosaccharides. In one study, an HILIC column with gradient elution using water and acetonitrile was employed, with an evaporative light scattering detector used for detection. This method successfully analyzed DP2-5 oligosaccharides [62]. Size-exclusion chromatography in HPLC is used not only for analyzing laminaran but also for determining its molecular weight distribution. The size-exclusion chromatograph system is calibrated using standard dextrans of known molecular weights, establishing a calibration curve by plotting their elution times against molecular sizes. By comparing the retention time of laminaran to this curve, its molecular weight can be estimated. For example, an Ultrahydrogel 2000 column was used to separate laminaran and determine its molecular weight range, which was found to be 5–46 kDa [63].

### 4.4. Enzymatic Assays

Enzymatic assays are a highly precise method for quantifying laminaran, utilizing the specificity of enzymes that target β-glucan linkages. These assays typically involve β-glucanase or laminarinase, which hydrolyze laminaran into smaller oligosaccharides or glucose units. The resulting hydrolysis products can then be quantified through secondary techniques such as HPLC or colorimetric glucose assays, including the glucose oxidase–peroxidase method [64].

Compared to traditional chemical approaches, enzymatic assays offer superior specificity as they selectively degrade laminaran without interference from other polysaccharides. Additionally, they exhibit high sensitivity, detecting even trace amounts of laminaran in complex biological or environmental samples. However, factors such as enzyme purity, reaction conditions, and substrate specificity must be carefully optimized to ensure accuracy and reproducibility. Becker et al. described the cloning and expression of three glycoside hydrolases: FaGH16A (GH16 family), FaGH17A (GH17 family), and FbGH30 (GH30 family). FaGH17A specifically cleaves the β-1,3-glucose backbone, whereas FbGH30 hydrolyzes β-1,6-glucose side chains and mixed-linked glucans. The use of these enzymes for laminaran quantification yielded results comparable to the conventional acid hydrolysis method but with enhanced speed and specificity. For instance, in *Thalassiosira weissflogii*, the enzymatic method measured 6–20 pg of glucan per cell, whereas acid hydrolysis yielded values of 8–23 pg per cell [65].

### 4.5. Fourier Transform Infrared Spectroscopy

Fourier transform infrared (FTIR) spectroscopy is widely utilized to examine the structural and functional groups of laminaran by measuring its infrared absorption across different wavelengths [66,67]. Laminaran typically displays a broad absorption band in the range of 3200–3400 cm^−1^, corresponding to O–H stretching vibrations, which signifies the presence of hydroxyl groups commonly found in polysaccharides [68]. The C–H stretching vibrations associated with sugar rings are observed between 2800 and 3000 cm^−1^ [69,70]. The fingerprint region (1200–900 cm^−1^) plays a vital role in detecting C–O–C and C–O–H stretching vibrations, as well as β-glucan structures. A distinguishing feature of laminaran is the absence of strong absorption peaks near 1740 and 1250 cm^−1^, indicating ester (C=O) groups [71]. Additionally, a pronounced band in the 890–910 cm⁻^1^ range confirms the existence of β-(1→3) glycosidic linkages, a defining trait of laminaran [72]. FTIR spectroscopy serves as a fast and valuable technique for the structural identification and validation of laminaran in carbohydrate chemistry, food science, and pharmaceutical research. Nonetheless, integrating FTIR with complementary methods such as nuclear magnetic resonance (NMR) and chromatography enables a more detailed structural analysis.

### 4.6. Mass Spectrometry

**Figure 3:**
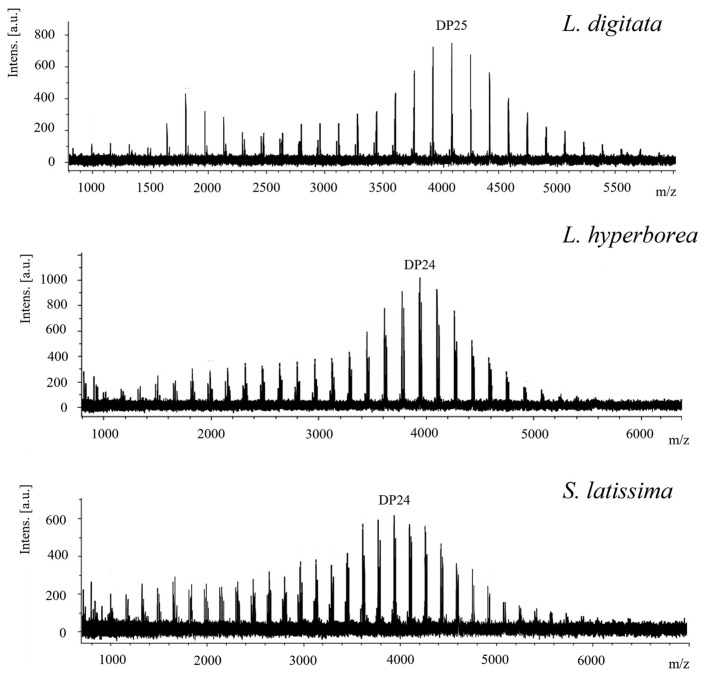
MALDI-TOF MS spectra of laminaran from *Laminaria digitata*, *Laminaria hyperborea*, and *Saccharina latissima*. Reproduced from reference [79] under terms of Creative Commons Attribution (CC-BY) license.

**Figure 4:**
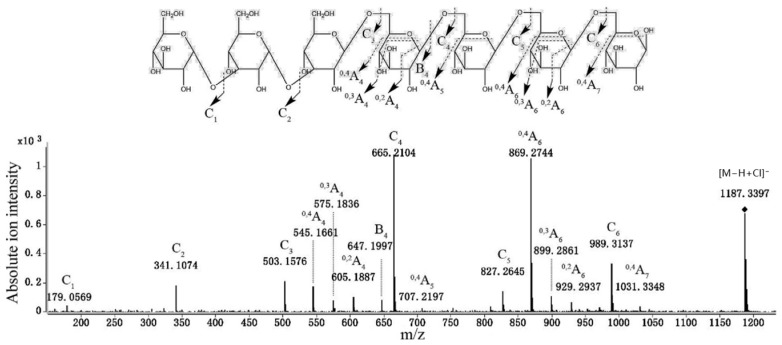
HPAEC-PAD-MS/MS analysis of laminaran hydrolysis products with a degree of polymerization of 7. The figure was adapted with permission from reference [80], copyright 2018, Elsevier.

Mass spectrometry involves determining the molecular weight, DP, and structural properties of laminaran. During mass spectrometry analysis, laminaran undergoes ionization and fragmentation, allowing for the identification of its molecular components based on their mass-to-charge ratios [73]. Two commonly used methods for polysaccharide analysis are matrix-assisted laser desorption/ionization time-of-flight mass spectrometry (MALDI-TOF-MS) and electrospray ionization mass spectrometry (ESI-MS) [74,75]. MALDI-TOF-MS is particularly effective in assessing the molecular weight distribution of laminaran and its oligosaccharides, whereas ESI-MS provides detailed fragmentation patterns that offer insights into glycosidic linkages and branching structures [76,77,78]. MALDI-TOF MS characterization of laminaran has demonstrated variations in the DP among different brown seaweed species, generally ranging from DP7 to DP36. For instance, *L. digitata* primarily contains DP25, while *Laminaria hyperborea* and *Saccharina latissima* predominantly exhibit DP24 (Figure 3) [79]. Furthermore, ESI-MS has been used to differentiate laminaran molecular weights, identifying values of 4300 Da for *L. hyperborea* and 2100 Da for *L. digitata* [30]. Tandem mass spectrometry (MS/MS) enables a deeper understanding of laminaran’s structural characteristics by fragmenting molecules into smaller components. Mass spectrometry is frequently combined with chromatographic methods, such as HPLC or high-performance anion exchange chromatography (HPAEC), with pulsed amperometric detection being used to enhance separation and analysis. For instance, the MS/MS analysis of laminaran hydrolyzed by recombinant endo-β-1,3-glucanase from *Coprinopsis cinerea* identified a DP7 hydrolysis product with cross-ring cleavage ions (^0,4^A, ^0,3^A, and ^0,2^A) appearing at the fourth and sixth residues from the non-reducing end, while ^0,4^A ions were detected at the fifth and seventh residues. These results suggest that the DP7 structure consists of Glc-(1→3)-Glc-(1→3)-Glc-(1→6)-Glc-(1→6)-Glc-(1→6)-Glc-(1→6)-Glc (Figure 4) [80].

### 4.7. Nuclear Magnetic Resonance Spectroscopy

Nuclear magnetic resonance (NMR) spectroscopy is an essential technique for determining the structural characteristics of laminaran, offering detailed information on its glycosidic linkages and anomeric configurations [81,82]. When paired with partially methylated alditol acetate derivatives, NMR enhances the identification of glycosidic linkages. Methylation analysis using partially methylated alditol acetate reveals which hydroxyl groups participate in glycosidic bonding, while NMR further clarifies their exact configurations [83]. Following methylation, the sample undergoes hydrolysis, reduction, and acetylation before being analyzed via gas chromatography–mass spectrometry (GC-MS) to determine the methylation patterns of individual sugar residues [84]. A glycosyl linkage analysis of methylated laminaran through GC-MS has identified specific diagnostic fragments, such as 1,5-di-*O*-acetyl-1-deuterio-2,3,4,6-tetra-*O*-methyl-glucitol, 1,3,5-tri-*O*-acetyl-1-deuterio-2,4,6-tri-O-methyl-glucitol, and 1,3,5,6-tetra-O-acetyl-1-deuterio-2,4-di-*O*-methyl-glucitol. These fragments confirm the presence of terminal non-reducing glucopyranosyl (t-Glcp), (1→3)-Glcp, and (1→3,6)-Glcp residues in laminaran [17].

Both proton NMR (^1^H NMR) and carbon NMR (^13^C NMR) are widely applied to examine laminaran’s structural properties. In ^1^H NMR spectra, anomeric proton signals typically appear between 4.5 and 5.5 ppm, indicating the presence of β-(1→3) and β-(1→6) glycosidic linkages. Meanwhile, ^13^C NMR provides further verification, with anomeric carbon signals detected between 100 and 105 ppm, which are characteristic of β-glucans. Specific anomeric region signals correspond to structural fragments as follows: →3)-β-D-Glcp-(1→3)- at 96.7/4.77 ppm; →6)-β-D-Glcp-(1→3)- at 103.9/4.69 ppm; →3)-β-D-Glcp-(1→6)- at 102.4/4.36 ppm; and β-D-Glcp-(1→6)- at 103.7/4.52 ppm [85,86].

**Figure 5:**
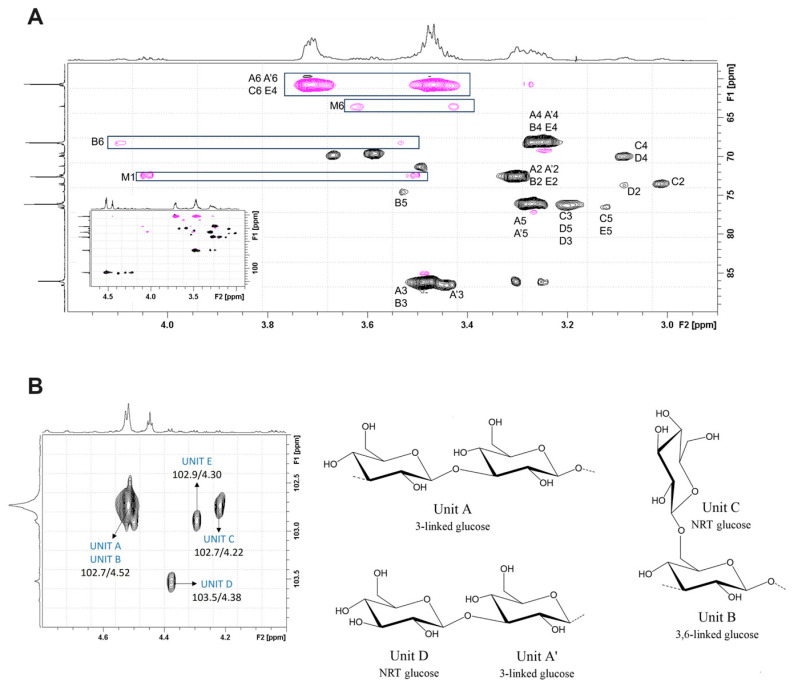
The anomeric region of the HSQC NMR spectra of laminaran (**A**), accompanied with a schematic representation (**B**) of its identified structural units. The figure was adapted with permission from reference [85], copyright 2025, Elsevier. Creative Commons CC-BY license.

Additional NMR techniques, including two-dimensional correlation spectroscopy (COSY), heteronuclear single quantum coherence (HSQC), and heteronuclear multiple bond correlation (HMBC), provide a more in-depth analysis of sugar unit connectivity and branching patterns [87,88]. In the selective HSQC spectrum of laminaran, four cross-peaks were detected at 102.7/4.52–4.53, 102.7/4.22, 103.5/4.38, and 102.9/4.30 ppm, with corresponding repeating unit structures illustrated in Figure 5A,B [85]. While NMR delivers highly precise structural data without the need for derivatization, it requires high-purity samples and adequate concentrations to achieve optimal resolution.

## 5. Biological Activities of Laminaran

### 5.1. Antioxidant Activity

**Figure 6:**
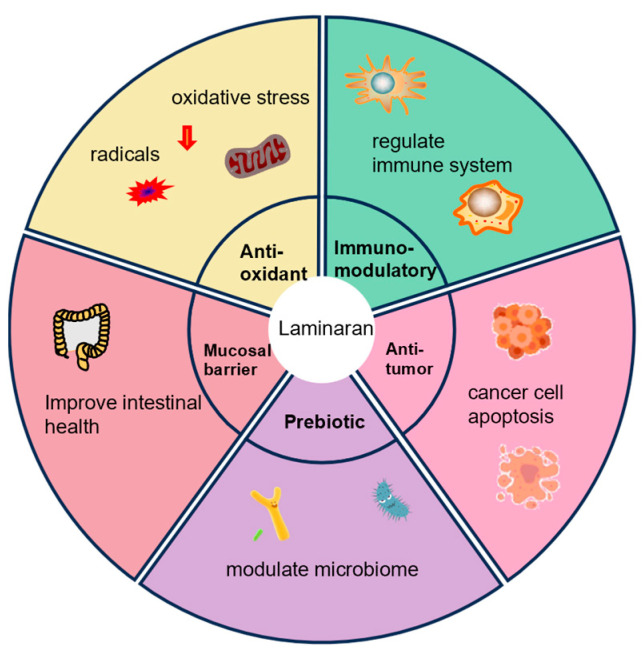
The diverse biological activities of laminaran, including antioxidant, immunomodulatory, antitumor, gut health-promoting, and prebiotic effects. The red downward arrows indicate a reduction in oxidative stress and the scavenging of radicals.

Laminaran exhibits diverse biological activities, including antioxidant, immunomodulatory, antitumor, gut health-promoting, and prebiotic effects (Figure 6). Antioxidant capacity represents the ability of a substance to counteract reactive oxygen species (ROS) and other oxidizing agents, thereby mitigating oxidative stress and protecting against cellular damage [89]. Oxidative stress occurs due to the imbalance between ROS generation and the body’s antioxidant defense mechanisms that neutralize these harmful molecules [90]. ROS, which encompass superoxide radicals, hydroxyl radicals, and hydrogen peroxide, are highly reactive compounds capable of oxidizing biomolecules such as lipids, proteins, and DNA, resulting in cellular dysfunction, inflammation, and tissue damage [91,92]. Laminaran has gained significant attention for its antioxidant properties, which are attributed to three primary mechanisms: free radical scavenging, metal ion chelation, and the modulation of enzymatic activity [42,93,94].

Free radicals are highly reactive molecules characterized by the presence of unpaired electrons, which contribute to their instability [95]. These molecules can induce oxidative stress by triggering chain reactions that compromise cellular components. Laminaran has hydroxyl or functional groups, which enhance its ability to donate electrons. This inherent property allows laminaran to effectively interact with free radicals and neutralize their harmful effects [96,97]. Laminaran functions as a free radical neutralizer by donating electrons to stabilize reactive species. For example, research has highlighted laminaran’s ability to significantly scavenge mitochondrial superoxide anions (O_2_^−^). In experiments using the rat gastric epithelial cell line RGM1, laminaran treatment induced by indomethacin resulted in a reduction in O_2_^−^ levels by 16.64%, while administration under dabigatran achieved a decrease of up to 54.80%. Furthermore, laminaran can indirectly hinder the formation of hydroxyl radicals (•OH) and peroxynitrite (ONOO^−^) by removing O_2_^−^, thereby reinforcing its potent antioxidant capabilities [98]. Laminaran oligosaccharides demonstrate reactivity with oxidizing agents such as 1,1-diphenyl-2-picrylhydrazyl and 2,2′-azino-bis(3-ethylbenzothiazoline-6-sulfonic acid) [62]. The ability of laminaran to scavenge these radicals is attributed to its hydrogen-donating capacity, which leads to the formation of stable end products.

Transition metal ions such as iron, copper, chromium, and cobalt are crucial contributors to oxidative stress via the Fenton reaction process [99]. Under physiological conditions, this reaction converts hydrogen peroxide into highly reactive hydroxyl radicals, which can damage cellular components and contribute to the development of various disease [100]. To alleviate this issue, natural antioxidants utilize metal chelation. Laminaran purified from *Cystoseira barbata* demonstrates an ability to reduce ferric ions and binds to these metal ions through interactions based on electrostatic forces and coordination bonds [101]. This binding process forms stable complexes that prevent the free diffusion of metal ions, thereby minimizing their availability for the Fenton reaction [102]. By reducing the catalytic activity of transition metal ions, laminaran offers a robust defense mechanism against oxidative stress.

The body’s antioxidant defense system also relies on intrinsic enzymes such as superoxide dismutase, catalase, and glutathione peroxidase [103]. Superoxide dismutase catalyzes the dismutation of superoxide radicals into hydrogen peroxide, which is then degraded into water and oxygen by catalase and glutathione peroxidase [104]. Collectively, these enzymes function as a protective mechanism to counteract oxidative stress and preserve cellular balance [105]. Ahn et al. explored the impact of laminaran on ultraviolet B-induced skin damage in an in vivo model. Their study demonstrated that ultraviolet B exposure significantly decreased the levels of superoxide dismutase, glutathione peroxidase, and catalase. However, laminaran treatment enhanced the expression of antioxidant enzymes, thereby strengthening the skin’s ability to combat ROS [106]. Moreover, dietary supplementation with 0.8% laminaran in spotted sea bass (*Lateolabrax maculatus*) led to an increase in glutathione levels, superoxide dismutase activity, and total antioxidant capacity in serum [107].

### 5.2. Immunomodulatory Effects

Laminaran plays a vital role in modulating innate immune cells, such as macrophages, dendritic cells, and natural killer cells, contributing to immune homeostasis and defense mechanisms. Macrophages, key players in the innate immune system, are capable of polarizing into either the pro-inflammatory M1 phenotype or the anti-inflammatory M2 phenotype in response to specific environmental signals [108,109]. In a lipopolysaccharide-induced acute lung injury mouse model, laminaran reduced the expression of CD86, a marker linked to M1 macrophages [26]. Additionally, Zhu et al. reported that laminaran enhances NK cell cytotoxicity in immunosuppressed mice [110].

The immunoregulatory properties of laminaran are mediated through interactions with pattern recognition receptors, which detect pathogen-associated molecular patterns and initiate immune responses. Among these receptors, Dectin-1 is a well-characterized β-glucan receptor predominantly found on macrophages, dendritic cells, and neutrophils [111,112]. Studies on murine RAW264.7 and human THP-1 macrophages demonstrated that laminaran functions as a Dectin-1 agonist, modulating TNF-α production. Silencing Dectin-1 in both RAW and THP-1 cells significantly diminished the TNF-α response to laminaran treatment, indicating that its biological activity is largely mediated via this receptor [113]. The interaction between laminaran and Dectin-1 activates intracellular signaling cascades, triggering phagocytosis, reactive oxygen species generation, and cytokine release, thereby enhancing innate immune responses. Additionally, laminaran engages Toll-like receptors (TLRs), particularly TLR4, which stimulates the NF-κB signaling pathway, leading to the transcription of pro-inflammatory cytokines [114,115]. Rattigan et al. demonstrated that dietary laminaran supplementation in pigs exposed to dextran sodium sulfate significantly modulated gene expression, including TLR4, MMP2, MMP1, IL6, and IL10 [116].

Laminaran also regulates cytokine production, playing a dual role in immune homeostasis. Under pathogenic conditions, it enhances the secretion of pro-inflammatory cytokines such as IL-6 and IL-1β to reinforce immune defense while promoting anti-inflammatory cytokines like IL-10 to mitigate excessive inflammation and tissue damage [117,118]. A study assessing laminaran and its oligosaccharide derivatives found that their effects on cytokine secretion varied. Specifically, dendritic cells treated with laminaran from *Laminaria hyperborea* exhibited a 24% increase in IL-6 secretion compared to the control. Conversely, laminaran-derived oligosaccharides from Saccharina latissima had no impact on IL-6, IL-12p40, or IL-10 secretion by dendritic cells. However, the smallest oligosaccharide fraction from Saccharina latissima led to an 18% reduction in TNF-α secretion, suggesting anti-inflammatory properties [79]. The immunomodulatory effects are primarily regulated through NF-κB, MAPK, and JAK/STAT signaling pathways [119,120]. The activation of the MAPK pathway modulates macrophage and dendritic cell responses, shaping the cytokine landscape. Similarly, laminaran’s influence on the JAK/STAT cascade enhances anti-inflammatory cytokine expression and regulatory immune factors, contributing to immune balance [121]. By engaging these pathways, laminaran not only aids in pathogen clearance and immune activation but also mitigates chronic inflammation and immune dysregulation.

Beyond innate immunity, laminaran also impacts adaptive immune responses by influencing T and B lymphocyte activity, thereby contributing to long-term immune protection [122]. It promotes T-helper (Th) cell differentiation, particularly enhancing Th1 and Th17 responses, which are essential for combating infections and suppressing tumor progression. Laminaran administration resulted in a marked expansion of CD25^+^Foxp3^+^ Treg cells in the colonic lamina propria. This expansion was associated with the suppression of colitis, suggesting that Treg cells play a crucial role in mediating the protective effects of laminaran [123]. Additionally, its ability to activate antigen-presenting cells, such as macrophages and dendritic cells, enhances adaptive immunity by improving antigen presentation and co-stimulatory signaling, leading to robust T and B cell responses. Broquet et al. explored the potential of laminaran to induce trained immunity, mimicking sepsis-induced immune reprogramming to counteract tumor growth. In their study, mice received intraperitoneal injections of laminaran before being challenged with either *Escherichia coli*-induced sepsis or Lewis lung carcinoma. The results showed that laminaran stimulated trained immunity in macrophages, increasing the tissue residency of CXCR6^+^ T cells and reducing tumor growth [124]. The protective effects of laminaran were dependent on resident alveolar macrophages as their depletion nullified its benefits. These findings underscore laminaran’s therapeutic potential in enhancing antitumor immunity by mimicking the immune reprogramming observed following sepsis [124].

### 5.3. Antitumor Activity

In contemporary oncological research, antitumor activity has emerged as a pivotal area of focus. This emphasis extends beyond the mere elimination of primary tumors to encompass strategies aimed at preventing their spread and recurrence. Among the diverse therapeutic approaches being explored, natural compounds, particularly those sourced from marine environments, have garnered significant attention. These compounds are valued for their varied mechanisms of action and favorable toxicity profiles [125,126]. Laminaran has gained considerable recognition due to its robust antitumor capabilities. Its effectiveness stems from a comprehensive strategy that includes inducing apoptosis, inhibiting angiogenesis, preventing metastasis, and modulating the immune response. This multifaceted approach underscores laminaran’s potential as a valuable tool in the fight against cancer.

One of the most striking features of laminaran’s antitumor activity lies in its capacity to trigger apoptosis in cancer or tumor cells [127]. By engaging both intrinsic and extrinsic pathways that orchestrate cell death, laminaran effectively curtails tumor growth and progression while minimizing harm to healthy cells. Experimental evidence has shown that laminaran exerts significant anti-proliferative effects and induces apoptosis in human hepatocellular carcinoma cells (Bel-7404 and HepG2) in a dose-dependent manner across concentrations of 15–35 mg/mL. Furthermore, in vivo investigations involving tumor-bearing mice (Hepa 1–6 model) have underscored laminaran’s ability to effectively suppress tumor growth [128]. In addition, laminaran has been demonstrated to progressively inhibit the proliferation of human ovarian cancer cells (ES2 and OV90) in a dose-dependent manner (0.1–2.0 mg/mL). This effect is primarily mediated through the suppression of cell proliferation and the modulation of key intracellular signaling pathways such as PI3K/MAPK. Notably, in vivo studies employing a zebrafish xenograft model have revealed that laminaran significantly reduces tumor formation by 48.5% in ES2 cells and 45.6% in OV90 cells when treated with a concentration of 2 mg/mL prior to injection into the zebrafish yolk sac [129].

Laminaran exhibits anti-metastatic activity by suppressing the migratory capacity of cancer cells. By targeting the motility properties of these cells, laminaran diminishes their ability to infiltrate adjacent tissues and establish secondary lesions. This mechanism is particularly significant given that metastasis represents a major cause of cancer-related fatalities. Moreover, laminaran inhibits the expression of matrix metalloproteinases (MMP), enzymes responsible for extracellular matrix degradation [130]. In numerous cancers, elevated MMP-2 and MMP-9 levels promote tumor cell invasion and dissemination, enabling malignant cells to escape the primary site and initiate secondary growths [131]. In an in vitro study, native laminaran and sulfated laminarans derived from *Fucus evanescens* demonstrated potent anti-metastatic effects against MDA-MB-231 cells, achieving inhibition rates of 86% for native laminaran and 94% for sulfated laminaran. These findings also highlight the concurrent suppression of MMP-2 and MMP-9 activity [132]. The sulfated derivative of laminaran derived from *Dictyota dichotoma* exhibits protective effects against X-ray-induced damage in JB6 Cl41 cells. Upon irradiation, cell proliferation in JB6 Cl41 decreased by 43%, whereas a reduction of 34% was observed in SK-MEL-28 cells compared to control conditions. Notably, at a low concentration (25 μg/mL), the sulfated laminaran derivative significantly potentiated the radiation-induced suppression of SK-MEL-28 cell migration by 31% relative to irradiation alone. This protective effect was linked to the suppression of MMP-2 and MMP-9 activity, along with reduced phosphorylation of the ERK1/2 signaling pathway [133].

Laminaran exhibits potent immunostimulatory effects by activating various immune cells, including macrophages, natural killer cells, dendritic cells, and T lymphocytes. In addition to this, laminaran significantly enhances the production and secretion of essential cytokines—such as interleukins, interferons, and tumor necrosis factors—which are crucial for coordinating an effective, specific immune reaction. These cytokines play a key role in amplifying cytotoxic activity, optimizing antigen presentation, and modifying the tumor microenvironment to hinder cancer progression. Laminaran derived from *L. digitata* demonstrates remarkable ability to enhance the maturation and antigen-presenting capacity of splenic dendritic cells. When these dendritic cells are activated by laminaran, they effectively present tumor-associated antigens to T cells, including transgenic OT-1 and OT-2 T cell subsets. OT-1 T cells, which recognize antigens via MHC class I molecules, are pivotal for inducing cytotoxic responses against tumor cells. On the other hand, OT-2 T cells, responsive to antigens presented by MHC class II molecules, contribute to helper functions that further enhance overall immune activation and adaptive immune response [134].

### 5.4. Mucosal Barrier

The gut microbiota is essential for maintaining overall health as it contributes to modulating immune function and supports the integrity of the intestinal barrier. A diverse and well-balanced microbial community maintains gut health and supports efficient nutrient absorption [135]. Research indicates that laminaran can modulate the gut microbiota composition, contributing to intestinal homeostasis and strengthening the mucosal barrier [136,137]. As a prebiotic, laminaran selectively encourages the proliferation of beneficial bacterial strains such as *Bifidobacteria* and *Bacteroides*. In a 24 h in vitro human fecal fermentation study, laminaran significantly boosted the abundance of *Bifidobacteria* (Δ8.3% of total bacteria) and *Bacteroides* (Δ13.8% of total bacteria) [138]. Additionally, species like *Bacteroides xylanisolvens*, *Bacteroides uniformis*, and *Bacteroides fingoldii* were found to ferment laminaran. The fermentation broth derived from these bacteria, when combined with laminaran, enhanced nitric oxide production in RAW264.7 murine macrophages [139]. Moreover, the in vitro fermentation of laminaran extracted from *Sargassum henslowianum* using human fecal microbiota resulted in a decline in *Haemophilus parainfluenzae* levels [17]. This opportunistic pathogen is known for colonizing mucosal surfaces and evading immune defenses, especially in immunocompromised individuals [140]. The observed reduction in *H. parainfluenzae* suggests that laminaran may play a protective role in gut health. Liu et al. found that laminaran plays a regulatory role in functional dyspepsia induced by iodoacetamide by influencing gut microbiota composition. Notably, laminaran contributed to restoring microbial equilibrium by adjusting the relative proportions of Bacteroidetes and Firmicutes [136].

The breakdown of laminaran in the colon is primarily facilitated by gut microbiota, particularly *Bacteroides*, which play a key role in its fermentation. *Bacteroides* species harbor a wide range of carbohydrate-active enzymes that enable them to efficiently degrade laminaran into fermentable components [141]. This process, carried out by *Bacteroidetes*, results in the generation of short-chain fatty acids (SCFAs), such as acetate, propionate, and butyrate [142]. These SCFAs act as essential metabolic mediators, promoting gut barrier integrity, modulating immune function, and providing energy for colonic epithelial cells [143]. Additionally, SCFAs help sustain a balanced gut microbiota by lowering the intestinal pH, suppressing the growth of harmful bacteria, and influencing host metabolism [144]. Beyond gastrointestinal health, SCFAs are involved in systemic metabolic regulation by modulating glucose homeostasis, lipid metabolism, and appetite through interactions with G-protein-coupled receptors (GPR41 and GPR43) [145]. Furthermore, they play a role in immune modulation by promoting anti-inflammatory cytokine production and enhancing mucosal immune responses [146]. A decline in SCFAs levels has been linked to gut dysbiosis, inflammatory bowel diseases, and metabolic disorders [147,148]. A study by Strain et al. utilizing an in vitro colonic fermentation model demonstrated that laminaran extracted from *Laminaria digitata* (sourced from Ireland) significantly increased the concentrations of acetic acid, butyric acid, and total SCFAs compared to cellulose at all time points [149]. Moreover, *Bacteroides intestinalis*, isolated from five-week-old male mice from the Institute of Cancer Research, was found to ferment laminaran, predominantly producing lactate, whereas *Bacteroides acidifaciens* primarily generated succinate [150]. Lactate plays a critical role in SCFA production, particularly as a precursor for propionate and butyrate, via microbial cross-feeding interactions [151]. It also contributes to gut health by regulating intestinal pH, inhibiting pathogenic bacteria, and strengthening mucosal barrier integrity [152]. Succinate, another key fermentation metabolite, functions as both an energy source and an intermediate in metabolic pathways [153]. Specific gut bacteria, such as *Prevotella* and *Propionibacterium*, can further convert succinate into propionate, highlighting its role in microbial metabolism [154].

Chronic inflammation in the colon is frequently associated with increased levels of pro-inflammatory cytokines, which play a key role in immune cell activation, the disruption of the epithelial barrier, and tissue injury [155,156]. Interestingly, the pre-administration of laminaran prior to the induction of dextran sulfate sodium-induced colitis in pigs led to a significant reduction in IL6 mRNA expression [157]. On the other hand, the secretory immunoglobulin A (sIgA) is the predominant antibody found in mucosal secretions and plays a vital role in immune defense across epithelial surfaces, including the gastrointestinal, respiratory, and urogenital tracts. Within the gut, sIgA has a dual function: it helps maintain immune equilibrium by neutralizing pathogens and shaping the composition of gut microbiota. However, under certain conditions, such as NOD2 deficiency, sIgA activity may contribute to inflammation. Research indicates that the absence of NOD2 leads to increased sIgA transport, resulting in the excessive internalization of IgA-coated bacteria and an amplified inflammatory response. Interestingly, laminaran has been shown to inhibit this transport, suggesting that the modulation of the sIgA pathway could serve as a potential therapeutic approach for managing inflammation in inflammatory bowel disease. This is particularly significant for individuals with NOD2 mutations, who face a heightened risk of developing severe Crohn’s disease [158].

## 6. Conclusions

This review highlighted significant advancements in the extraction, characterization, and bioactive potential of laminaran. Extraction and purification techniques have evolved considerably over time, with innovative methods such as UAE, MAE, and EAE offering superior yields and purity compared to traditional methods. These advancements are essential for ensuring high-quality laminaran suitable for a wide range of applications in pharmaceuticals, nutraceuticals, and biomedical sciences. Moreover, the characterization of laminaran through various analytical techniques such as HPLC, MS, and NMR has facilitated a deeper understanding of its structural properties. These techniques provide detailed insights into the molecular weight, degree of polymerization, branching configurations, and functional groups, which are critical to its bioactivity and overall effectiveness. The bioactive potential of laminaran, particularly its antioxidant, immunomodulatory, antitumor, and mucosal barrier-enhancing activities, underscores its promise in diverse health applications. As research continues to unfold, laminaran’s potential for treating chronic diseases, modulating immune responses, and providing mucosal protection in the gastrointestinal tract remains an exciting area of exploration. Despite the promising findings, several research gaps remain in the study of laminaran. The first gap is the need for the development of standardized methods for extraction, purification, and characterization, with variability in reported results often being linked to different extraction methods and seaweed sources. In addition is the need for more in-depth studies into the mechanistic pathways underlying its bioactivity. While the antioxidant and immunomodulatory properties of laminaran are well documented, the molecular mechanisms by which it exerts its effects are still not fully understood due to limited in vivo studies that evaluate the long-term effects and safety profile of laminaran in humans. While preclinical studies demonstrate its efficacy, there is still a need for robust clinical trials to confirm its therapeutic benefits and establish optimal dosages for human consumption.

Future studies of laminran should focus on bridging the gap between laboratory-based research and clinical applications by conducting comprehensive in vivo and clinical trials. There is a growing interest in exploring the potential of laminaran in emerging fields, such as personalized medicine, where its antioxidant and immunomodulatory effects could be leveraged in targeted therapies. In addition, exploring laminaran’s interactions with other bioactive compounds could further enhance its therapeutic potential. Laminaran can be incorporated into various biomaterials, such as hydrogels, nanoparticles, and films, to create controlled-release systems for therapeutic agents. These systems enable the gradual release of drugs, nutrients, or bioactive compounds over a specified period, thereby enhancing efficacy and minimizing treatment side effects. Furthermore, ongoing research is expected to explore the molecular mechanisms underlying laminaran’s biological activities, offering deeper insights into its interactions with cellular pathways and its potential as a functional ingredient in food, cosmetics, and pharmaceuticals. With ongoing research and technological progress, laminaran has the potential to become a vital component in the future of functional biopolymers and marine-derived therapeutics.

## Data Availability

No new data were created or analyzed in this study. Data sharing is not applicable to this article.

## Abbreviations

DP: degree of polymerization; HWE: hot water extraction; UAE: ultrasound-assisted extraction; MAE: microwave-assisted extraction; EAE: enzymatic-assisted extraction; TLC: thin-layer chromatography; HPLC: high-performance liquid chromatography; HILIC: hydrophilic interaction liquid chromatography; FTIR: Fourier transform infrared; MALDI-TOF: matrix-assisted laser desorption/ionization time-of-flight; ESI-MS: electrospray ionization mass spectrometry; HPLC: high-performance liquid chromatography; HPAEC: high-performance anion exchange chromatography; NMR: nuclear magnetic resonance; GC-MS: chromatography–mass spectrometry; COSY: correlation spectroscopy; HSQC: heteronuclear single quantum coherence; HMBC: heteronuclear multiple bond correlation; ROS: reactive oxygen species; Th: T-helper; MMPs: matrix metalloproteinases; SCFAs: short-chain fatty acids; GPR: G-protein-coupled receptors; sIgA: secretory immunoglobulin A.
